# The influence of the Great East Japan earthquake on microscopic polyangiitis: A retrospective observational study

**DOI:** 10.1371/journal.pone.0177482

**Published:** 2017-05-12

**Authors:** Yoichi Takeuchi, Ayako Saito, Yoshie Ojima, Saeko Kagaya, Hirotaka Fukami, Hiroyuki Sato, Ken Matsuda, Tasuku Nagasawa

**Affiliations:** Division of Nephrology, Department of Medicine, Japanese Red Cross Ishinomaki Hospital, Ishinomaki, Miyagi, Japan; Tokushima University Graduate School, JAPAN

## Abstract

**Background:**

Antineutrophil cytoplasmic antibody-associated vasculitis is triggered by environmental factors, including silica dust exposure. Repeated tsunami waves brought a large volume of silica-containing sludge inland after the Great East Japan earthquake in 2011. We aimed to determine if the serious disaster influenced the clinical features of the microscopic polyangiitis.

**Methods:**

This is an observational retrospective study conducted in a single institute. A total of 43 patients were included based on the CHCC2012 criteria for microscopic polyangiitis from 2007 to 2015. We used the Poisson regression model to determine the incidence of microscopic polyangiitis within the annual population of the medical district. The participants were selected during a 3-year period from before (N = 13) to after the disaster (N = 20). The differences of parameters and the overall survival between the groups were analyzed.

**Results:**

The incidence of microscopic polyangiitis increased after the disaster (λ = 17.4/million/year [95%CI: 7.66–39.6] before the disaster and λ = 33.1/million/year [17.7–61.7] after the disaster, *P* = 0.044). A high Birmingham Activity Score was associated with a high incidence of microscopic polyangiitis after the disaster. The overall survival of the patients with microscopic polyangiitis declined significantly after the disaster.

**Conclusions:**

The Great East Japan earthquake influenced the development of the microscopic polyangiitis in our restricted area. The patients who developed after the disaster had severe symptoms and a high mortality rate.

## Introduction

The Great East Japan earthquake (GEJE) occurred at 14:46 JST on Mar 11 in 2011 with a magnitude of 9.0, which was the most powerful earthquake on record in Japan. Large tsunamis hit the pacific coastal areas of Northeast Japan about 30 min after the earthquake. In harbor cities in particular, there was catastrophic loss of life and infrastructure due to the enormous waves [1]. Over 18,000 people were reported dead or missing.

A large number of survivors was evacuated and forced to stay in emergency shelters under poor hygienic conditions due to overcrowding. The survivors in these shelters were exposed to dusty micro-particles scattered from the sludge, which contained marine bacteria and toxic materials brought inland by the tsunamis. Even after relocating to temporary housing, most of the residents continued to encounter long-standing health problems. Inactivity-mediated chronic diseases such as sleeplessness, dementia, and aspiration pneumonia were promoted by the breaking of social and familial ties. It was actually reported that the numbers of community-acquired pneumonia and bronchial asthma increased after the GEJE due to prolonged poor sanitation in the emergency shelters or temporary housing [2].

Antineutrophil cytoplasmic antibody (ANCA)-associated vasculitis can be triggered by silica dust exposure and bacterial and viral infections [3]. Silica exists in the natural environment within soil, cement, and window glass. The outbreak of myeloperoxidase (MPO)-ANCA-associated vasculitis (AAV) was noted within the 3-year period following the Hanshin-Awaji earthquake in 1995 in Japan, which was characteristic of the severe collapses of many buildings containing asbestos and silica [4]. Similarly, in the aftermath of the GEJE, the sludge brought inland by the tsunamis was found to contain a large amount of silica dust. Here, we focused on the influence of the GEJE on the patients with microscopic polyangiitis (MPA), which is predominant in Japan.

In the coastal area of Northeast Japan, there are many rural cities that have a considerable population of the elderly. Among the damaged municipalities in Japan, Ishinomaki city had the greatest number of victims and the highest number of temporary housing during this massive disaster. Our hospital played a leading role in accommodating various patients involved in the disaster because it was located close to the center of the flooded area but was not directly damaged by the tsunamis.

The aim of this study was to examine whether the GEJE influenced the clinical features of MPA in this medical district. To achieve this, we retrospectively analyzed the characteristics of MPA based on information gathered from medical records.

## Materials and methods

### Selection of participants

**Fig 1:**
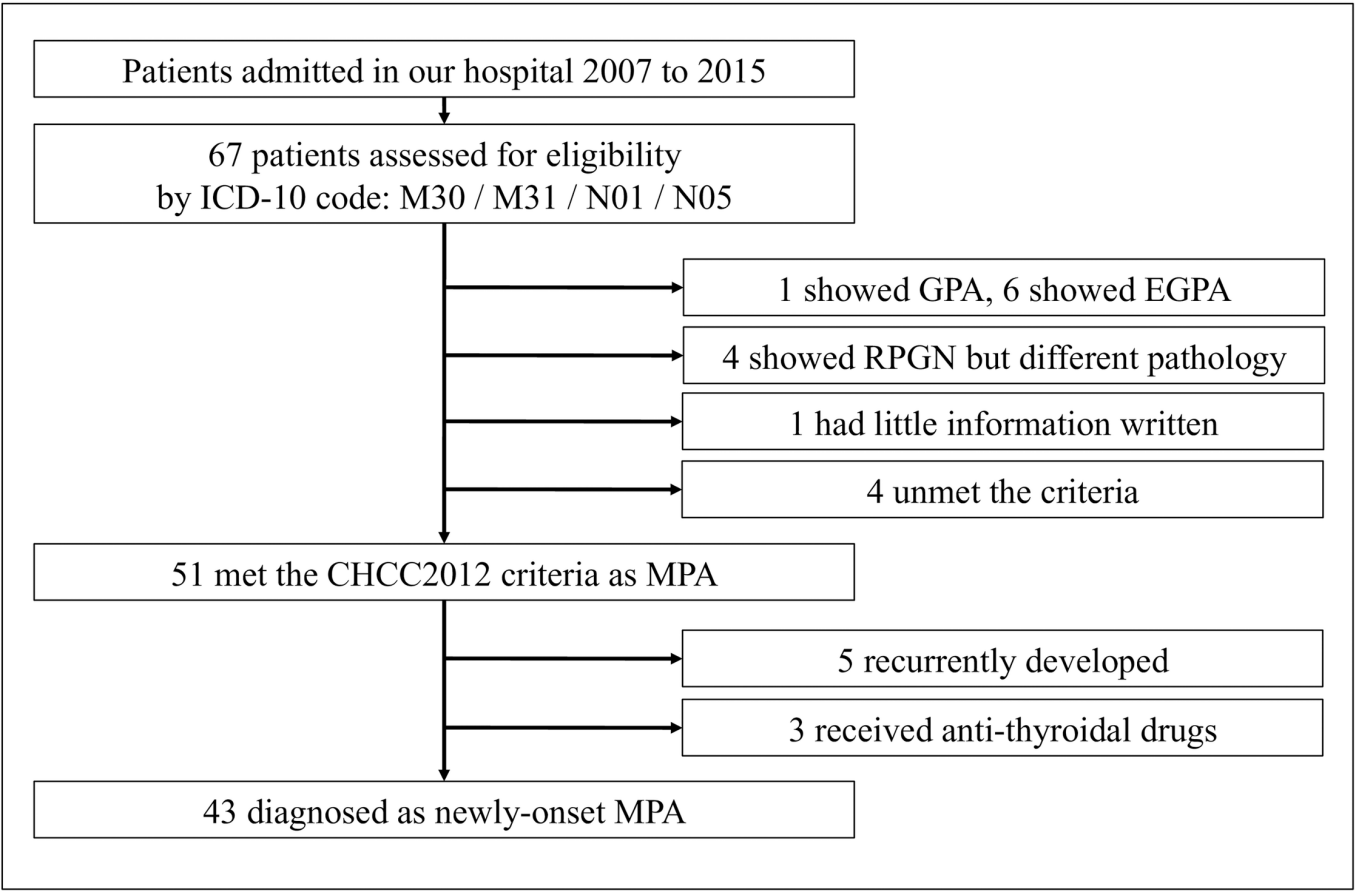
Flow diagram of screening for participants in this study. The crude number of patients with microscopic polyangiitis was counted on the basis of ICD-10 codes from medical records in our hospital. Each of the patients was diagnosed with MPA based on the CHCC2012 criteria. Drug-induced and recurrent cases were excluded. Abbreviations: ICD, international classification of diseases; RPGN, rapidly progressive glomerulonephritis; GPA, granulomatosis with polyangiitis; EGPA, eosinophilic granulomatosis with polyangiitis, CHCC; Chapel Hill Consensus Conference, MPA; microscopic polyangiitis.

This study was a descriptive case-series study performed in a single core medical center. The patients in whom MPA developed were selected according the flow diagram in **Fig 1**. We precisely selected patients admitted between March 2007 and March 2015 registered with the ICD-10 codes M30, M31, N01, and N05 from the medical records at our hospital. The participants were included based on the criteria of the Chapel Hill Consensus Conference 2012 [5]; eligibility was assessed by three clinical nephrologists at our hospital. As our hospital is often visited by many patients living in and near Ishinomaki City who are referred by their family clinicians, the cooperation of these clinicians enabled us to acquire information relating to participants’ coexisting diseases and their sequential serum creatinine levels before visiting our institution. Using these records, we defined rapidly progressive glomerulonephritis (RPGN) as a > 30% increase in the serum creatinine level occurring over several weeks to three months accompanied by both, microscopic hematuria and proteinuria [6]. We performed a renal needle biopsy for a precise diagnosis if a patient showed signs of RPGN. All the referred patients underwent computed tomography at presentation to detect pulmonary alveolar hemorrhage or interstitial pneumonitis. Professional radiologists at our hospital evaluated all the images. The four patients who unmet the CHCC criteria (in **Fig 1**) showed serological ANCA-positivity without clinical RPGN pattern or being subjected to renal biopsies, which were not even suspicious for any vasculitis. Besides, we excluded patients who met the following criteria: MPA relapse in the study period (inaccuracy in clinical diagnosis) or vasculitides triggered by antithyroid agents (difference of medical therapy or prognosis). Baseline characteristics and the Birmingham Vasculitis Activity Scores (BVASs) of the participants were obtained from medical records on the day of admission. Respiratory symptom was counted as the combined measures of respiratory discomfort and/or cough. The term “coexisting disease” like CKD or hypertension was defined as each past medical illness stated clearly in each medical record or in the referral letters. Using serum creatinine levels (measured with enzymatic methods) and age, we calculated the estimated glomerular filtration rate (eGFR) according to the formula recommended by the Japanese Society of Nephrology [7]. The ANCA titer was determined by using commercially available ELISA kits. The values of the ANCA titers were categorized as mild (< 50IU/ml), intermediate (> 50 IU/ml, < 200 IU/ml), or high (> 200 IU/ml) concentrations.

To ensure that all medical information was of high quality, we started the enrollment of MPA patients since April 2007, when the medical records at our institute were computerized. The participants were enrolled in the study on the date of their first visit and classified into two groups on 11^th^ March 2011 based on whether they developed MPA before or after the GEJE.

### Statistical analysis

The count data of rare events are generally described as a Poisson distribution. In order to elucidate the incidence of MPA before and after the disaster, the number-of-event endpoints were compared between study groups during the 4-year period before and after the GEJE using a Poisson regression model [8]. First, we defined the categorical variables before and after 11^th^ March 2011 and assigned a valued of 1 or 0, which indicated whether or not the sample was observed in the corresponding period. Then, we fitted the Poisson regression model with an offset parameter of the annual population of the Ishinomaki medical district as shown in **S1 Fig**. As a candidate value for the annual population, we used the value of the population in September among those monthly reported by the Miyagi Prefectural Government. We analyzed newly diagnosed MPA cases and excluded recurrent cases because the recurrent events failed to meet the rule of the Poisson distribution, “events occur independently.”

Furthermore, we examined the factorial variables between the two groups to elucidate the clinical characters of the patients developing the MPA during the relatively acute phase of the disaster. In order to observe the participants for more than two years in the subsequent time-to-event analysis, we selected the participants during a 3-year period from before (13 people, March 2008 to February 2010) to after the GEJE (20 people, March 2011 to February 2013). The differences between the two groups were analyzed by using Fisher’s exact test for categorical valuables and the two-tailed Mann-Whitney *U* test for numerical values at a significance level of 0.05.

Overall survival was calculated from the date of their admissions to the date of death or data censoring (for the patients who remained alive on March 2015 and those who dropped out) according to the Kaplan-Meier method, basing the comparison on the log rank test.

All statistical data were analyzed by using EZR (Saitama Medical Center, Jichi Medical University, Saitama, Japan), which is a graphical user interface for R version 3.2.1 (The R Foundation for Statistical Computing, Vienna, Austria) [9].

This work was not supported by any foundation. The institutional review board (IRB) of the Japan Red Cross Ishinomaki Hospital permitted us to analyze the medical records of the patients included in the study (IRB approval number 14–19).

## Results

A total of 43 MPA patients were included in this population-based study. Baseline patient characteristics at presentation are given in **Table 1**. The median age was 74 years, and 44% of the participants were women. With respect to coexisting diseases, hypertension was found in 58% of the patients, chronic kidney disease in 25%, and diabetes mellitus in 12%. The primary initial symptoms at presentation were fever > 37.2°C (21%) and respiratory discomfort (16%). The median values of serological data were as follows: hemoglobin concentration, 8.9 g/dL; serum creatinine level, 1.90 mg/dL; and C-reactive protein level, 4.80 mg/dL. Half of the patients had a high MPO-ANCA titer (> 200 IU/mL). Approximately 60% of participants had ground-glass opacities in the lungs as seen on computed tomography. RPGN was diagnosed in about 80% of the patients. Renal biopsies for precise diagnosis were performed in 44% of the patients. In all patients who underwent renal biopsies, we verified typical histological findings of necrotizing glomerulonephritis with crescent formation. The median value of BVAS was 18 points.

**Table 1:** Baseline characteristics of 43 MPA patients in this study.

Characteristics	
Age (years)	74.00 [68.00, 80.50]
Sex (female)	19 (44.2%)
Body Mass Index (kg m^-2^)	20.80 [17.62, 24.50]
Coexisting diseases	
Hypertension	25 (58.1%)
Chronic kidney disease	10 (25.0%)
Diabetes Mellitus	5 (12.2%)
Initial symptoms	
Slight fever	9 (20.9%)
Respiratory discomfort	7 (16.3%)
Cough	7 (16.3%)
Fatigue	6 (14.0%)
Pretibial edema	5 (11.6%)
Laboratory data on admission	
White Blood Cell (10^3^/μL)	8.80 [7.65, 11.40]
Hemoglobin (g/dL)	8.90 [7.70, 10.75]
Creatinine (mg/dL)	1.90 [1.22, 3.85]
eGFR (mL/min/1.73mm^2^)	22.95 [12.32, 37.63]
CRP (mg/dL)	4.80 [0.81, 11.07]
MPO-ANCA (U/mL)	
high (>200)	22 (51.2%)
intermediate (<200, >50)	11 (25.6%)
mild (<50)	10 (23.3%)
Ground glass opacity of chest CT	20 (60.6%)
RPGN	34 (81.0%)
Renal biopsies performed	19 (44.1%)
BVAS	18.00 [15.00, 21.50]

Each parameter listed was extracted from medical records at our hospital. The results are presented as numbers with percentages (%) for qualitative data and medians with IQRs (25^th^ percentile to 75^th^ percentile) for quantitative data. Abbreviations: MPA, microscopic polyangiitis; CRP, C-reactive protein; IQR, interquartile range; MPO-ANCA, myeloperoxidase antineutrophil cytoplasmic antibody; RPGN, rapidly progressive glomerulonephritis; BVAS, Birmingham Vasculitis Activity Score. “Conversion factors for units: serum creatinine in mg/dL to umol/L, x 88.4.”

**Fig 2:**
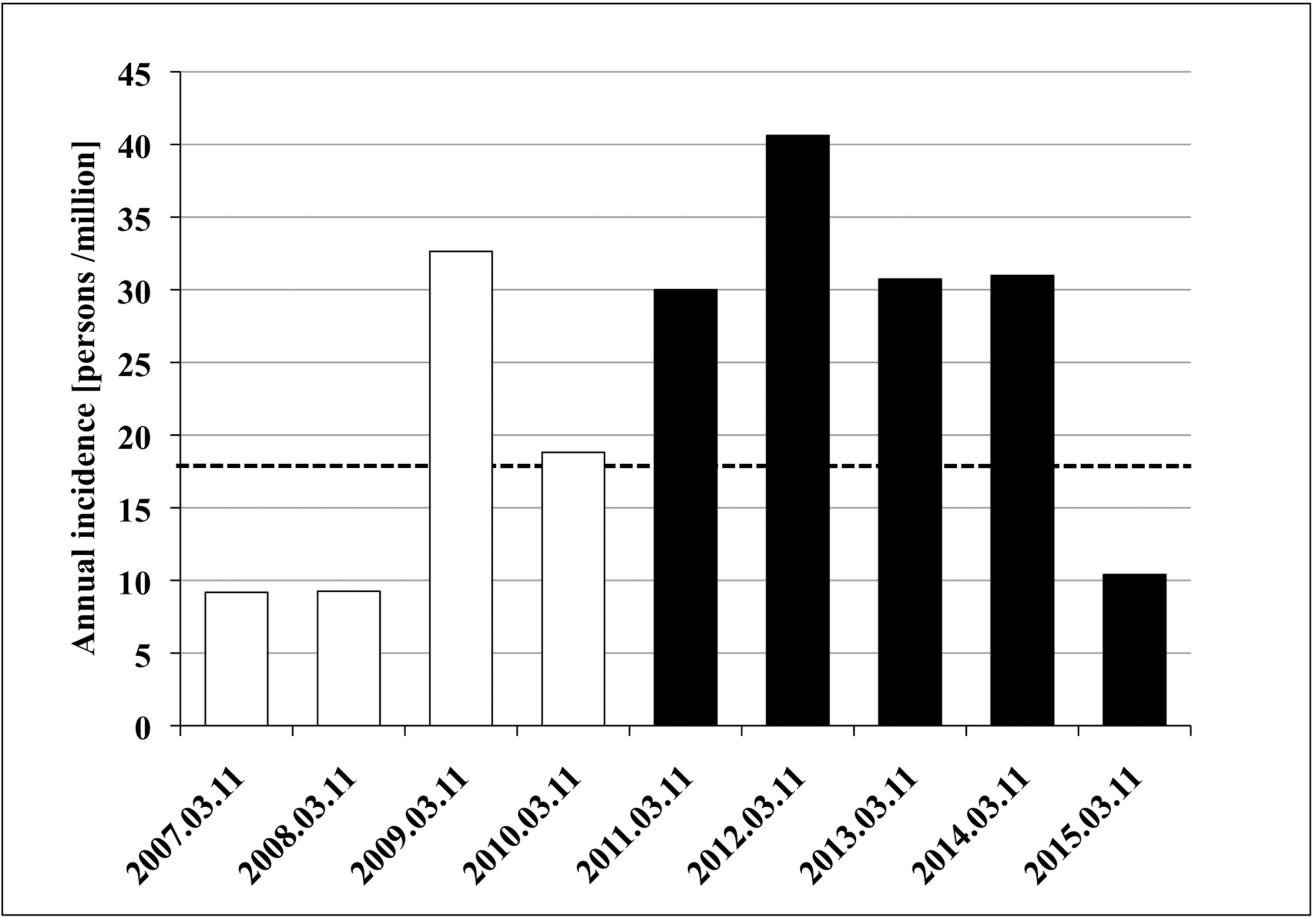
Change in the annual incidence of microscopic polyangiitis (MPA) in the Ishinomaki medical area. The incident cases were divided by the population at risk in the restricted medical area and plotted according to annual intervals. The white and black bars represent the incidence per 1 million people before and after the disaster, respectively. The mean incidence of MPA was 17.4 /million/year [95% Confidence Interval (CI): 7.66 to 39.61] before the disaster and 33.1 /million/year [95% CI: 17.73 to 61.74] after the disaster. The dashed line indicates the mean annual incidence of MPA in Japan. The incidence ratio and *P*-value were obtained using Poisson regression analysis (*P* = 0.044).

The incidence of MPA was defined as the proportion of the number of events to the population at risk in the Ishinomaki medical district. The results are presented in **Fig 2**. The mean incidence of MPA before and after the GEJE were 17.4 /million/year [95% Confidence Interval (CI): 7.66 to 39.61] and 33.1 /million/year [95% CI: 17.73 to 61.74], respectively. According to the Poisson regression model, the annual incidence doubled after the GEJE (*P* = 0.044). As the mean annual incidence of MPA is typically 18.2 /million people in Japan, the annual incidence after the GEJE in this area was well above the Japanese average [10].

Next, we compared the demographic and serological data of the MPA patients after the GEJE with those before the GEJE. The results are presented in **Table 2**. We detected significantly higher BVAS in the participants after the GEJE. C-reactive protein levels, age, and body mass index were likely to be higher in the patients who developed MPA after the GEJE than in those who developed MPA before the GEJE. The eGFR on admission tended to be lower in those who developed MPA after the GEJE. Compared to those who developed MPA before the GEJE, those with MPA after the GEJE were less likely to have hypertension or diabetes mellitus, but these differences were not statistically significant (*P* = 0.31 for hypertension, *P* = 0.63 for diabetes mellitus). The proportion of respiratory symptom was, although it was not significant, increased after GEJE (*P* = 0.132). Though the population of high MPO-ANCA titer seemed to decrease after GEJE, the difference in the two groups was not significant (*P* = 0.29).

**Table 2:** Comparison of factors before and after the GEJE.

	Before GEJE	After GEJE	
Variables	(n = 13)	(n = 20)	*P* value
Age (years)	73.00 [66.00, 75.00]	77.00 [68.00, 81.25]	0.1
Sex (female)	8 (61.5%)	7 (35.0%)	0.2
Body Mass Index	20.30 [18.00, 22.38]	22.20 [18.62, 25.55]	0.2
Coexisting diseases			
Hypertension	9 (69.2%)	10 (50.0%)	0.3
Chronic kidney disease	3 (27.3%)	5 (26.3%)	1
Diabetes Mellitus	2 (16.7%)	2 (10.5%)	0.6
Respiratory symptom	2 (15.4%)	9 (45.0%)	0.1
Laboratory data on admission			
Total protein (g/dL)	6.70 [6.27, 7.03]	6.50 [6.05, 7.30]	0.8
White Blood Cell (/μL)	8.40 [7.50, 9.40]	8.80 [7.62, 11.25]	0.6
Hemoglobin (g/dL)	10.00 [7.30, 10.70]	8.80 [7.37, 11.03]	1
Creatinine (mg/dL)	1.50 [1.10, 1.90]	2.67 [1.28, 4.88]	0.2
eGFR (mL/min/1.73mm^2^)	26.86 [20.35, 41.11]	17.16 [9.91, 34.64]	0.2
CRP (mg/dL)	3.90 [0.30, 8.90]	9.13 [1.39, 15.12]	0.1
MPO-ANCA			0.3
high	9 (69.27%)	9 (45%)	
intermediate	3 (23%)	5 (25%)	
mild	1 (7.7%)	6 (30%)	
Ground glass opacity of chest CT	7 (53.8%)	13 (65.0%)	0.7
Pulmonary alveolar hemorrhage	3 (23.1%)	4 (20.0%)	1
RPGN	9 (69.2%)	16 (80.0%)	0.7
BVAS (points)	16.00 [12.00, 18.00]	18.00 [16.00, 22.00]	**0.02**
Prognosis (death)	4 (30.8%)	12 (60.0%)	0.2

We compared the parameters of 13 MPA patients before the GEJE with those of 20 patients after the GEJE observed during 3-year periods surrounding the disaster. The results are presented as numbers with percentages (%) for qualitative data and medians with IQRs (25^th^ percentile to 75^th^ percentile) for quantitative data. Fisher’s exact test was applied for categorical valuables and a non-parametric Mann-Whitney *U*-test was applied for numerical valuables. Abbreviations: GEJE, Great East Japan earthquake; MPA, microscopic polyangiitis; CRP, C-reactive protein; IQR, interquartile range; MPO-ANCA, myeloperoxidase antineutrophil cytoplasmic antibody; RPGN, rapidly progressive glomerulonephritis; BVAS, Birmingham Vasculitis Activity Score. “Conversion factors for units: serum creatinine in mg/dL to umol/L, x 88.4.”

**Fig 3:**
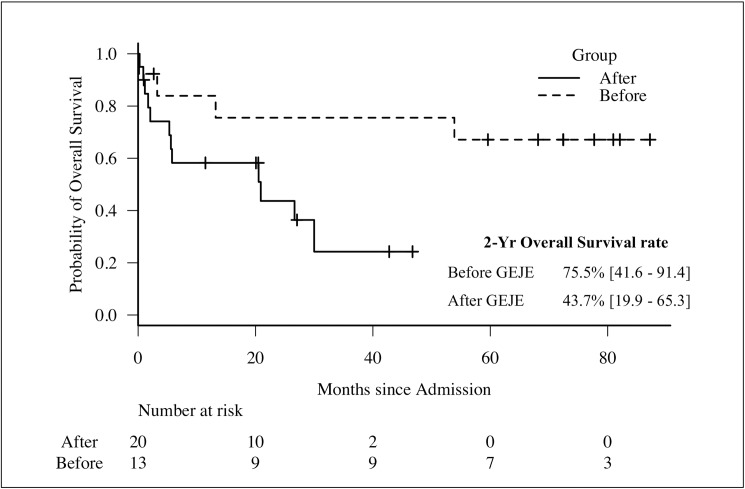
Comparison of overall survival between before and after the disaster. Survival curves were plotted to compare the mortality of each group using the Kaplan-Meier method. The dotted and solid lines indicate the time-to-events of participants before and after the disaster, respectively. The notches within the line represent the censors. The numbers at risk are illustrated below the chart. Overall survival was significantly worse in the group after the disaster (*P* = 0.029 by log rank test).

Finally, we examined the prognoses of the patients using the Kaplan-Meier method. The results are shown in **Fig 3**. The primary endpoint of all-cause death occurred in four patients during the three years before the GEJE and 12 patients after the GEJE (**Table 2**). The estimated 2-year overall survival rates were 75.5% [95%CI: 41.6 to 91.4] in the participants who developed MPA before the GEJE and 43.7% [95%CI: 19.9 to 65.3] in those who developed MPA after the GEJE (*P* = 0.029 by log rank test).

## Discussion

This study aimed to examine whether the earthquakes and tsunamis influenced the clinical features of MPA. We observed a considerable number of the patients with MPA after the GEJE, which was a similar phenomenon to the Hanshin-Awaji earthquake in 1995 [4]. We found that patients with MPA after the GEJE were likely to be older and high BVAS. Furthermore, the mortality rate of these patients with MPA during the critical period was much higher than that of the patients before the disaster.

Primary systemic vasculitis has been associated with exposure to silica, asbestos, hydrocarbons, and heavy metals [11]. In convincing epidemiological studies, especially, silica dust is investigated as the risk of AAV. Hogan *et al*. demonstrated that high levels of lifetime silica exposure increased the risk of AAV onset in a population-based study [12]. An important biologic evidence of silica exposure in the development of MPO-AAV has also been growing. Dostert *et al*. demonstrated that the inhalation of asbestos or silica activated the innate immune system through the induction of the Nalp3 inflammasome, whose subsequent activation led to the proinflammatory cytokine interleukin-1b secretion [13]. It is reported that the disordered regulation of neutrophil extracellular traps (NETs) may be closely related to the pathogenesis of MPA [14]. The NET is also one of the innate immune systems that capture and kill microorganisms and is produced by primed neutrophils, activated by proinflammatory cytokines. The serum DNaseI usually digests the NET debris immediately; however, the function can be partially abrogated in some conditions. Nakazawa *et al*. demonstrated that DNaseI activity in sera from Japanese patients with MPO-AAV was significantly lower than that in healthy control [15]. It suggested that impaired degradation of NETs was critically involved in the generation of MPO-ANCA. In addition, the genetic contribution to AAV has also been investigated. While PR3-AAV has the convincing association with the HLA DPB1*0401 in Caucasian [16], MPA and MPO-AAV, which are predominant in the Japanese, have the pathogenic association with DRB1*09:01 [17] and the protective association with DRB1*13:02 [18]. Namely, the survivors with the biologic and genetic susceptibility for MPO-ANCA production might be likely to develop MPA when inhaling the immuno-reactive microparticles like silica.

We have two questions to be discussed here. First, why did the number of MPA patients change after this disaster? The earthquake and subsequent tsunami brought more than 12 million cubic meters of marine sludge and sediments in the flooded area of the disaster. The marine sludge was composed primarily of sand and fine-grained soil from the seabed, in addition to microorganisms, organic hydrocarbons, heavy metals, and chemical compounds such as dioxins and poly-vinyl chloride [19]. Plenty of fine particles drifting on the air faced to the survivors who were engaged in the removal of the sludge outside or in the repair of mud-covered houses. In fact, in the neighboring coastal prefecture, Fukuhara *et al*. reported the transient increase of ANCA-positive interstitial pneumonia after GEJE [20]. Interestingly, after the GEJE, the bronchoalveolar lavage of the patients with the pulmonary hemorrhage contained the silica concentration much more than those before GEJE [21]. Yamanda *et al*. reported two restoration workers of the organizing pneumonia secondary to the inhalation of the dried marine sludge in the GEJE. An electron probe microanalysis of the lung specimens allowed confirmation of the presence of the oxidized silica in the inflammatory lesions. The authors also commented the atmospheric silica particle could reach the peripheral zone of the lung [22]. To our knowledge, although no data exists on measuring the atmospheric concentration of silica following GEJE, it is feasible to think that even the relatively healthy elderly had many chances to inhale the immuno-reactive fine particles like silica, and some of them resulted in developing MPA.

Other remaining factors should be considered about the increased number of MPA. The survivors felt deep sorrow and anxiety due to the breaking of social and familial ties. Such mental stress may have induced the neutrophil activation, which triggered the ANCA production [23]. For other factors, socio-economical services (many volunteers and caseworkers from all around Japan for the handicapped evacuees, medical-care-free doctrine for all evacuees by the Japanese government) may transiently facilitate the access to the hospital for the elderly, and that may result in increasing the potential patients with MPA. Nevertheless, such medical aid and comfort were insufficient for most of the evacuees. In order to rule out the confounding factors other than silica exposure and to highlight the association between silica exposure and development of MPA, evaluating the contemporary incidence of other diseases is needed. We examined the incidence of IgA nephropathy, one of general nephritides, as a control during the disaster. The result is illustrated in **S2 Fig**. The incidence of the IgA nephropathy after GEJE became similar to that before GEJE. It implies that environmental factors except for silica were irrelevant to the development of MPA.

The second question that needs to be answered is why the mortality of patients with MPA increased after the disaster. In a nationwide survey, cumulative overall survival at 1-year was 81.3% among Japanese patients with AAV [24], and the average age of the AAV onset was 66.8 years. Here, we should consider that the GEJE hit the aging cities of Northeast Japan, where the elderly comprised over a quarter of their total population. Bomback *et al*. demonstrated that elderly patients with MPA had a severer mortality rate than the young patients [25]. Here in **Table 1**, we noticed the MPA patients developed after the GEJE were older than those before the GEJE. The older age of MPA patients enrolled after the GEJE may explain the poorer prognoses than that of those enrolled before the GEJE. The other reason may be the inadequate control of co-morbid diseases. Many evacuees were forced to take high-salt preserved foods during a critical period and to get very less physical activities in the narrow temporary housing [26, 27]. Yamanouchi *et al*. also elucidated that one of the main causes of preventable disaster deaths was deteriorated environmental conditions in homes and emergency shelters at the coastal area in the GEJE [28]. In another original cohort study, Takahashi *et al*. revealed that the evacuees living in the temporary housing in the tsunami-stricken area had a significant increase in body weight due to the inconvenient condition for their life style or psychosocial state [29]. We speculated that most of the elderly participants were already medically uncontrolled in the pre-hospital phase, and they would have a disrupted immune system due to the long-standing stressful situations following the disaster. Such the inadequate health condition might be reflected by the slight gain observed in body mass index after the disaster in **Table 2**. Although we tried to use multivariate regression analysis for detecting the prognostic predictors of MPA after the GEJE, the sample size in this study was too small for an accurate analysis.

It would be important to evaluate our results with reference to previous articles. Yashiro *et al*. discussed the phenomenon of an increased number of MPA after the Hanshin-Awaji earthquake in 1995 [4]. They compared the clinical features of MPO-AAV patients living in the affected area (Kobe) with that of those living in distant areas. The MPO-AAV patients living in Kobe demonstrated higher C-reactive protein levels and white blood cell counts and more severe decline in kidney function. And more, a high rate of severe pulmonary involvement was characteristic, reflecting the localized intensive exposure of silica dusts owing to the building collapses. All of these findings correspond well with the results of our study. However, the mortality rate increased significantly after the GEJE in our study compared with that in the previous one. As far as we know, there have been no reports that silica exposure itself influenced the mortality of MPA patients. While the median age of the participants in Kobe in their report was 65.9 years, the median age of those after this disaster was 77.0 years as shown in **Table 2**. Therefore, the older age at enrollment of the MPA patients may explain the poorer prognoses found in our study. As a further example, Farquhar *et al*. detected no difference in the incidence or clinical manifestations of AAV after the Christchurch earthquake (magnitude 6.1) of New Zealand in 2011. For explaining the difference in these results compared to those from the previous report on the Hanshin-Awaji earthquake, the authors referred to the following factors: the lower population density, shorter study period, lower incidence of the MPO- versus the PR3-ANCA-associated vasculitis or less earthquake severity [30]. The GEJE, unlike the regional earthquake in Kobe or Christchurch, was unique in both, that tremendous tsunamis evoked by the underwater earthquake damaged the broader area of the aging coastal cities and that the origin of the silica particles would be derived mostly from the marine sludge.

We also obtained unexpected results. First, the number of patients with MPA transiently increased in 2009 as shown in **Fig 2**, which might be explained by the previous earthquake that occurred in the neighboring medical district (the Iwate-Miyagi Nairiku earthquake on June 14^th^ 2008 with a magnitude of 6.9). After the previous earthquake, the huge landslide in the mountains exposed the residents in the vicinity to a large amount of silica dusts. This suggests that huge earthquakes cause MPO-AAV. Second, the population of high MPO-ANCA titer seemed to decrease after GEJE, as shown in **Table 2**. There is no doubt that ANCA is important as a diagnostic marker. The value of measuring ANCA titer to predict disease activity or relapse, however, is controversial [31].

This study has several strengths and limitations. AAV is regarded as a rare autoimmune disease. Although it generally takes a long time to complete clinical cohort studies on AAV with sufficient sample sizes [32], our hospital, which was the only functioning medical institute in the area, happened to treat many MPA patients within a short period. We had the advantage of being able to count the precise number of MPA cases in the fixed medical district without the patients dispersing as previously reported [33]. However, the translocation or socioeconomic information of each participant for the epidemiological analysis could not be fully examined retrospectively from each medical record. Besides, we could not totally exclude the observer bias during the enrollment of participants. It is a debatable point that previous learning from the Hanshin-Awaji earthquake might allow us to screen the MPO-ANCA titer and to diagnose the vasculitis for many outpatients visiting our hospital after this disaster. Furthermore, in this small-sized study, we decided not to use a Cox regression model for time-to-event analysis because this study design might violate the rule of the proportional-hazards assumption with time independency. For example, in some cases, the participants enrolled before the GEJE may die or be censored during the aftermath of the disaster.

Japan, belonging to the Ring of Fire in the Pacific Ocean, is famous for being one of the most earthquake-prone areas in the world. Even after the GEJE, intense earthquakes have occurred in Kumamoto in Japan (Apr 16, 2016; magnitude 7.0), in Ecuador (Apr 16, 2016; magnitude 7.8), and in Taiwan (Feb 6, 2016; magnitude 6.4). Our study will be helpful for adequate planning and the precautions to be taken for the next major disaster.

In conclusion, we examined if the GEJE influenced the clinical features of MPA. The participants enrolled after the disaster had severe symptoms and a high mortality rate. We believe that this retrospective observational study will be an important milestone in providing new evidence of ANCA-associated vasculitis.

## Supporting information

S1 Appendix Clinical dataset of all the participants. (XLSX)

S2 Appendix Clinical dataset for the comparison of the pre- and post- disaster. (XLSX)

S1 Fig The dynamic change in the population of the Ishinomaki medical district. The change in the number of inhabitants in the Ishinomaki medical district during 2011 is illustrated by monthly intervals.
(TIF)

S2 Fig Change in the annual incidence of IgA nephropathy in the Ishinomaki medical district. The incident cases were divided by the population at risk in the restricted medical area and plotted according to annual intervals. The white and black bars represent the incidence per 1 million people before and after the disaster, respectively. The gray area indicates the estimated annual incidence of MPA in Japan [34].
(TIF)
