# Supplementary figures and images for: The influence of the Great East Japan earthquake on microscopic polyangiitis: A retrospective observational study
Source: PLoS One. 2017 May 12;12(5):e0177482. doi: 10.1371/journal.pone.0177482 (PMC5428958; doi:10.1371/journal.pone.0177482)

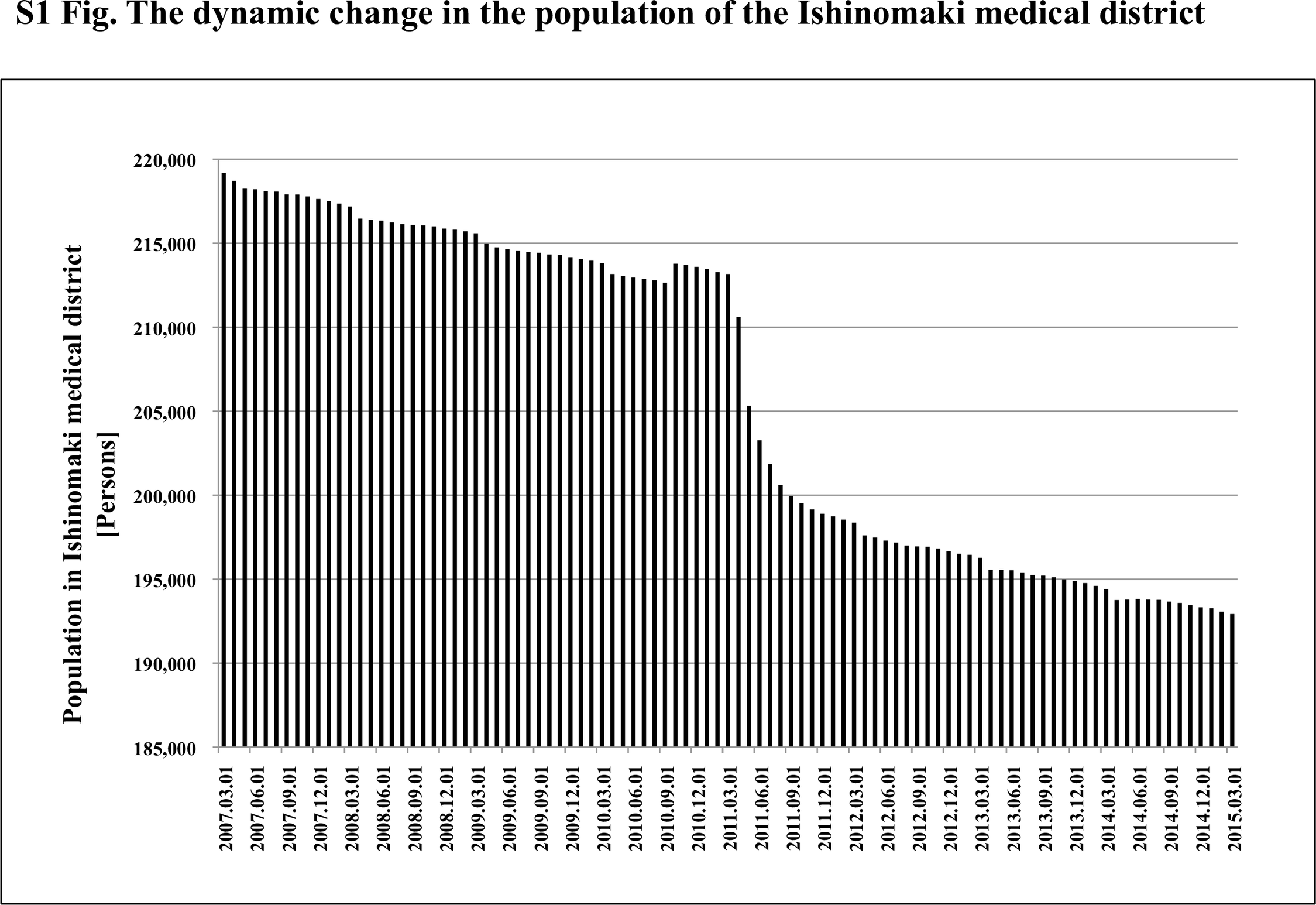

Supplement: S1 Fig — The change in the number of inhabitants in the Ishinomaki medical district during 2011 is illustrated by monthly intervals. (TIF) [file pone.0177482.s003.tif]

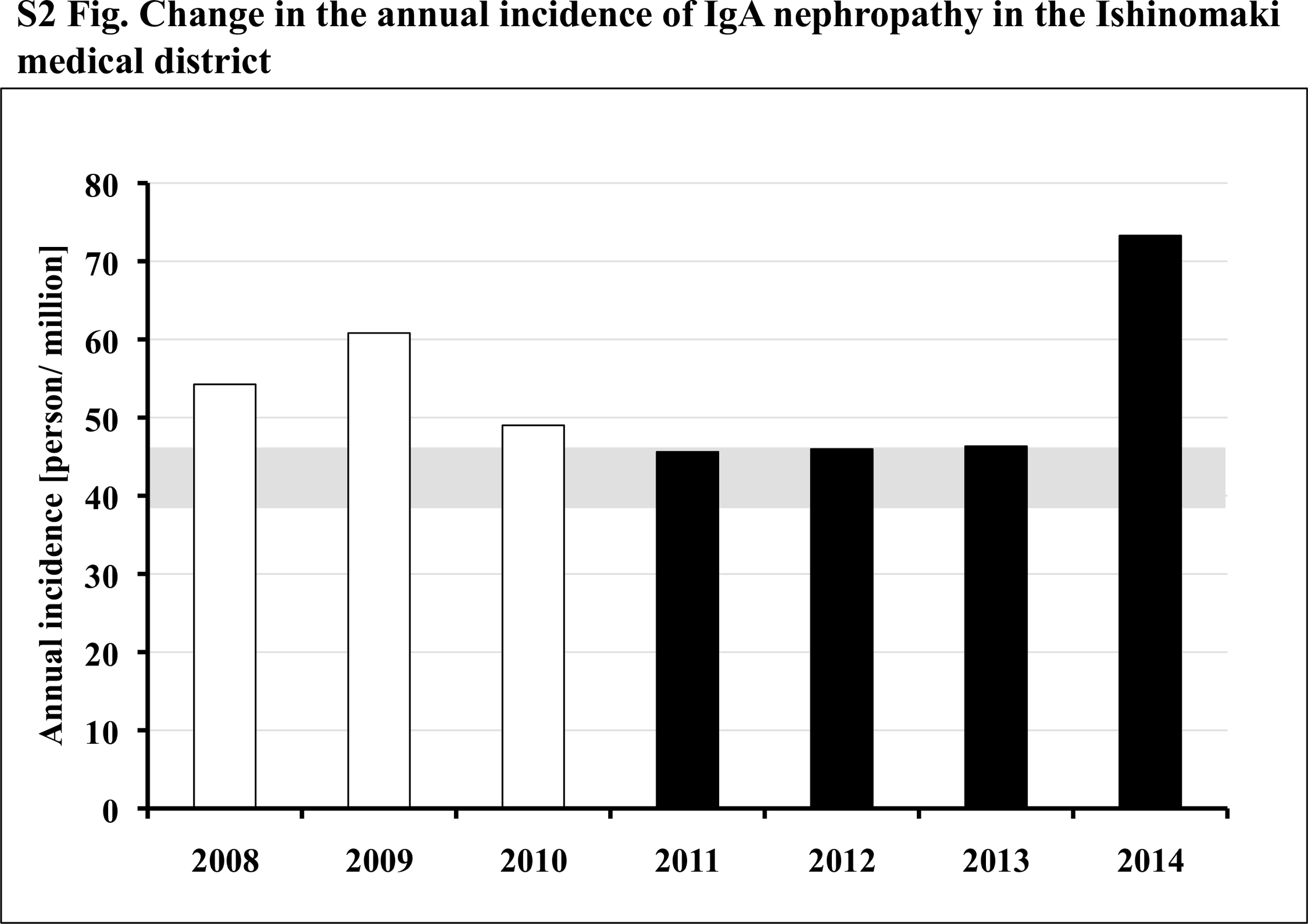

Supplement: S2 Fig — The incident cases were divided by the population at risk in the restricted medical area and plotted according to annual intervals. The white and black bars represent the incidence per 1 million people before and after the disaster, respectively. The gray area indicates the estimated annual incidence of MPA in Japan [34]. (TIF) [file pone.0177482.s004.tif]
